# Reconstruction of Intima and Adventitia Models into a State Undeformed by a Catheter by Using CT, IVUS, and Biplane X-Ray Angiogram Images

**DOI:** 10.1155/2017/9807617

**Published:** 2017-01-05

**Authors:** Jinwon Son, Young Choi

**Affiliations:** School of Mechanical Engineering, Chung-Ang University, 221 Heukseok-dong, Dongjak-gu, Seoul 156-756, Republic of Korea

## Abstract

The number of studies on blood flow analysis using fluid-structure interaction (FSI) analysis is increasing. Though a 3D blood vessel model that includes intima and adventitia is required for FSI analysis, there are difficulties in generating it using only one type of medical imaging. In this paper, we propose a 3D modeling method for accurate FSI analysis. An intravascular ultrasound (IVUS) image is used with biplane X-ray angiogram images to calculate the position and orientation of the blood vessel. However, these images show that the blood vessel is deformed by the catheter inserted into the blood vessel for IVUS imaging. To eliminate such deformation, a CT image was added and the two models were registered. First, a 3D model of the undeformed intima was generated using a CT image. In the second stage, a model of intima and adventitia deformed by the catheter was generated by combining the IVUS image and the X-ray angiogram images. A 3D model of intima and adventitia with the deformation caused by insertion of the catheter eliminated was generated by matching these 3D blood vessel models in different states. In addition, a 3D blood vessel model including bifurcation was generated using the proposed method.

## 1. Introduction

Thanks to the advances in computing and analysis techniques, studies on circulatory diseases using CFD (computational fluid dynamics) analysis are now using FSI (fluid-structure interaction) analysis that can take into account the movement of blood vessel walls [1–4]. As opposed to CFD analysis that requires only a 3D model of intima, a 3D model containing information about the blood vessel thickness is required to analyze the blood flow inside the blood vessel and the forces applied to the blood vessel wall using FSI analysis [5–9]. However, there are difficulties in generating a 3D model of blood vessel that includes both the intima and adventitia using only a single type of medical image, owing to the characteristics of the imaging techniques. To achieve this, many studies have proposed 3D modeling methods of blood vessels that include intima and adventitia by combining different imaging techniques or by making assumptions. The representative imaging techniques used for such blood vessel modeling methods are CT (computed tomography) and IVUS (intravascular ultrasound).

Though the shape of a blood vessel intima can be easily obtained using a CT image, no information can be obtained about the blood vessel adventitia. In addition, owing to the low accuracy of CT images, the blood vessel intima model generated using a CT image will have an uneven surface. Antiga has proposed a method of calculating the centerline of the blood vessel model to resolve the problem of the blood vessel model generated using such a CT image [10, 11]. A 3D blood vessel model generated using a CT image was automatically corrected using the centerline of the 3D blood vessel model, and a 3D blood vessel model that includes the intima and adventitia was generated with the assumption that the thickness of a blood vessel wall is proportional to the inner diameter.

An IVUS image is an image that shows the cross section of a blood vessel by inserting a microminiaturized ultrasonic instrument into the blood vessel. As an IVUS image is taken around a blood vessel, a lot more detailed information about the blood vessel can be obtained than that with a CT image. In addition, because an ultrasonic wave is used, it has the advantage that the information about the blood vessel adventitia can also be obtained. However, an IVUS image only shows the cross section of a blood vessel without showing the position and direction at which the IVUS image is taken. Whale has proposed a sequential triangulation method that calculates the position and orientation of an IVUS image using biplane X-ray angiogram images [12–17]. The 3D path along which IVUS images were taken was generated using biplane X-ray angiogram images, and the positions and orientations of these IVUS images were calculated using only the geometric shape of this path.

**Figure 1:**
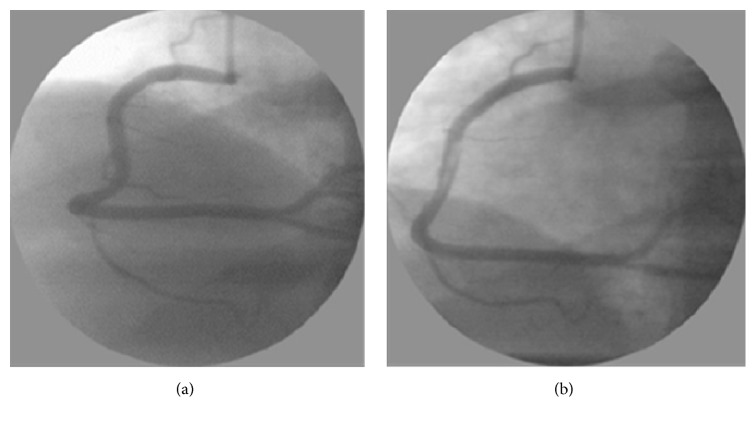
(a) Shape of blood vessel before catheter insertion. (b) Shape of blood vessel after catheter insertion [19].

However, the catheter inserted to take the IVUS image heavily deforms the blood vessel, as shown in Figure 1. Accordingly, the IVUS image and the biplane X-ray angiogram images taken with the catheter inserted show the information about the blood vessel deformed by the catheter insertion. In addition, the blood vessel model generated by combining these images will also be in a deformed state.

The initial state of the blood vessel has a great effect on the analysis result of the blood vessel model. Accordingly, in this study, we propose a 3D modeling method of intima and adventitia with the deformation caused by insertion of a catheter eliminated for accurate FSI analysis.

## 2. Overview

**Figure 2:**
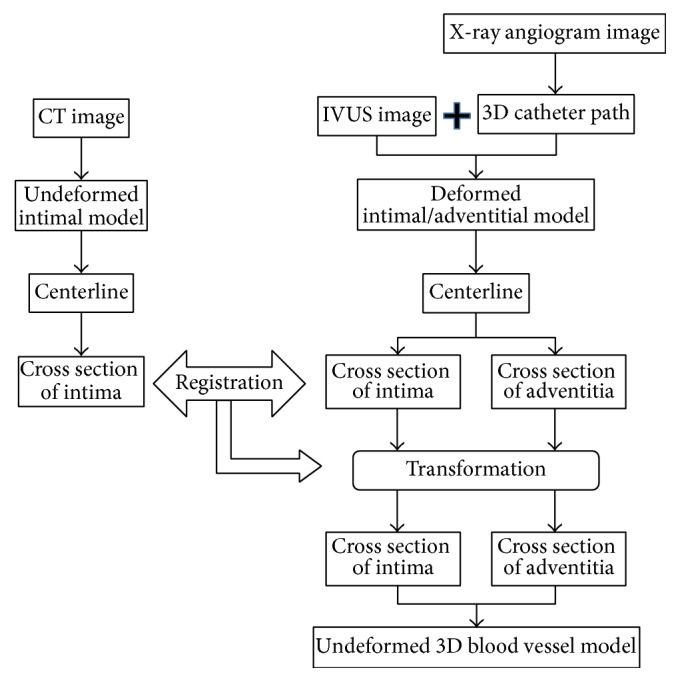
Overview of the proposed blood vessel modeling method.

Figure 2 shows the overall flow of the 3D blood vessel modeling method proposed in this study.

This method can be largely divided into three stages.

First, a 3D model of the undeformed intima is generated using a CT image. CT images only require a contrast medium to be administered and thus do not have any deformation caused by insertion of a catheter.

Then, a 3D intima and adventitia model in a state deformed by a catheter is generated by combining IVUS and biplane X-ray angiogram images. As explained earlier, IVUS images and biplane X-ray angiogram images show information about the blood vessel in a state deformed by insertion of a catheter.

The last stage involves converting the 3D model of the deformed intima and adventitia into a 3D model of the undeformed intima and adventitia through registration.

The last stage involves converting the 3D model of the deformed intima and adventitia into a 3D model of the undeformed intima and adventitia through registration. For this, the cross sections of the 3D models are extracted and registered. First, as the intimae exist in different states, the deformed intima is registered with the undeformed intima. The cross sections of the undeformed intima and adventitia are calculated by applying the registration result to the cross section of the deformed adventitia.

**Figure 3:**
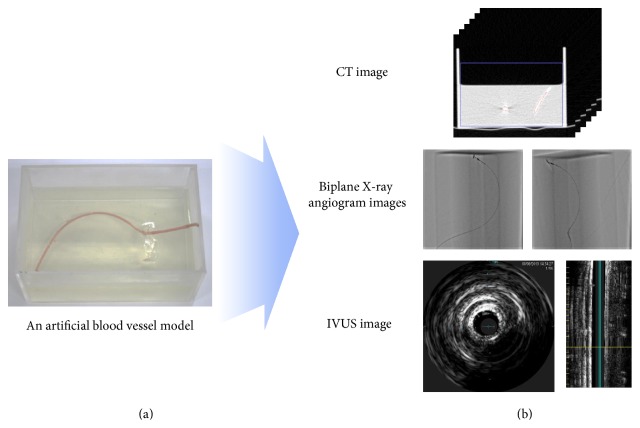
(a) Blood vessel replica. (b) CT, IVUS, and biplane X-ray angiogram images of the replica.

A blood vessel replica was produced as shown in Figure 3(a) to facilitate acquisition of the medical images required for the method proposed in this paper. A silicone tube was used as the replica blood vessel and gelatin was used to fix it and to enable it to be deformed when a catheter was inserted. Figure 3(b) shows the CT, IVUS, and biplane X-ray images taken using the blood vessel replica.

## 3. Reconstruction of Undeformed Intima Model

A CT image can be obtained without inserting a catheter into the blood vessel by administering a contrast medium and thus shows the undeformed shape of the blood vessel. However, as it only shows the contrast medium passing through the blood vessel, no information about the adventitia of the blood vessel can be acquired. Accordingly, we intended to utilize the overall shape of the blood vessel without the catheter-induced deformation by using such characteristics of CT images. For this, a 3D intima model with no catheter-induced deformation was generated using the CT image of the blood vessel replica.

**Figure 4:**
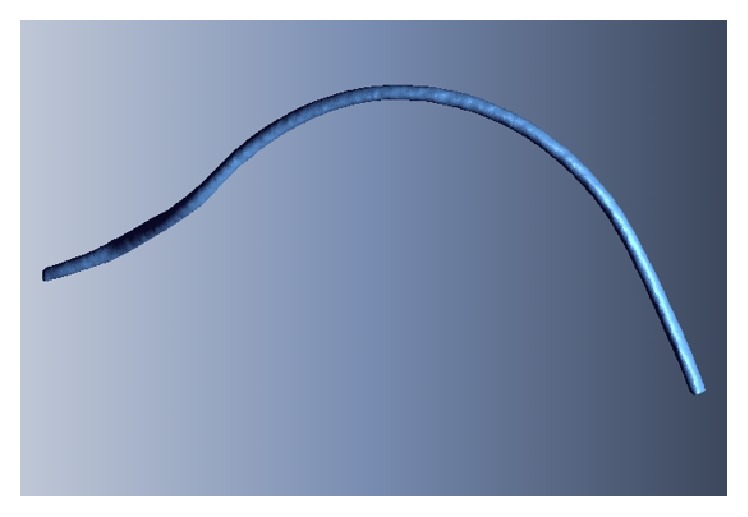
Generated 3D undeformed intima model of replica.

A CT image consists of voxel data produced by stacking tomograms of a human body. To generate a 3D blood vessel model, a process of extracting the polygon data corresponding to the blood vessel from the voxel data is required. To generate the polygon data of the blood vessel from the voxel data, the isosurfaces that have the same intensity value as that of the section corresponding to the blood vessel were extracted from each tomogram. A polygon model of the blood vessel was generated by stacking these isosurfaces and approximating the NURB surfaces.  Figure 4 shows the 3D model of the undeformed intima generated using the CT image of the blood vessel replica.

## 4. Reconstruction of Deformed Intima and Adventitia Model

### 4.1. Extraction of the Blood Vessel Intima and Adventitia Cross Sections in a Deformed State

**Figure 5:**
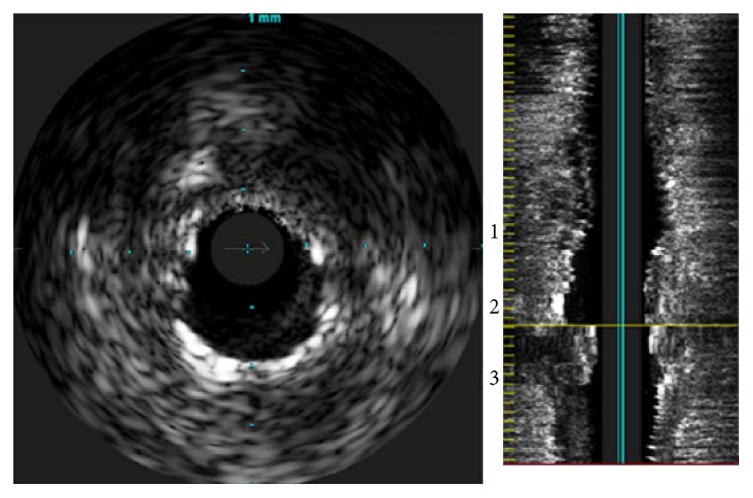
IVUS image of blood vessel.

An IVUS image shows the inside of a blood vessel in greater detail than a CT image as it is obtained by imaging the inside of the blood vessel with an ultrasonic device inserted into the blood vessel. Moreover, it provides information about the shape of the blood vessel adventitia.  Figure 5 shows the cross sections of blood vessel intima and adventitia extracted from an IVUS image.

**Figure 6:**
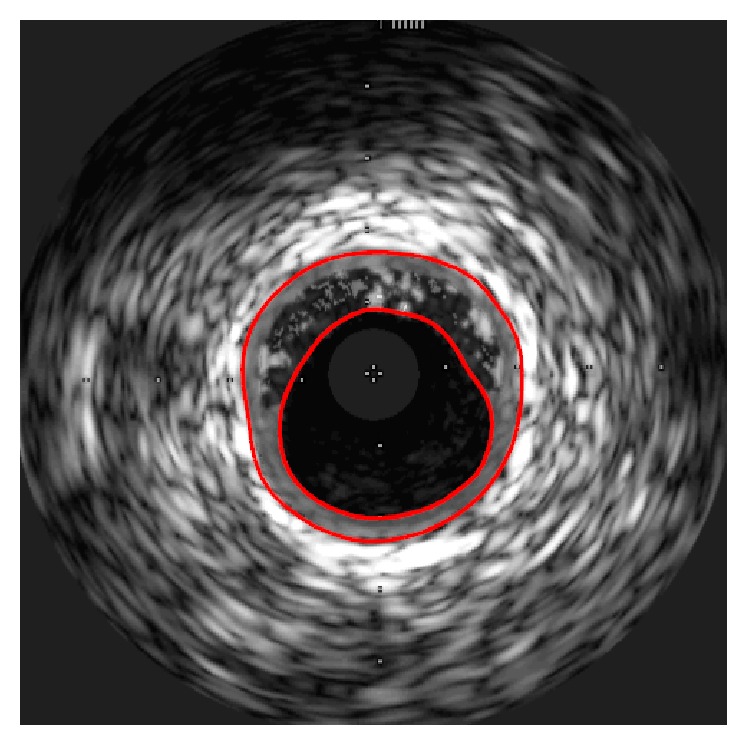
Segmented intima and adventitia contours from IVUS image.

As an IVUS image does not include color values but has points with gray scale values, there are difficulties in automatically extracting the areas corresponding to the intima and the adventitia of a blood vessel. Accordingly, in this study, we checked the IVUS image and manually segmented the sections corresponding to the intima and adventitia of the blood vessel, respectively, as shown in Figure 6.

### 4.2. Restoration of 3D Catheter Path

Though an IVUS image contains information about cross section of a blood vessel, the position and orientation at which the image was acquired are unknown. Accordingly, to generate a 3D blood vessel model using the cross sections of the blood vessel intima and adventitia extracted earlier from an IVUS image, the position and orientation where the IVUS image has been actually taken should be conjectured using other medical imaging techniques. For this, biplane X-ray angiogram images were used in this study.

**Figure 7:**
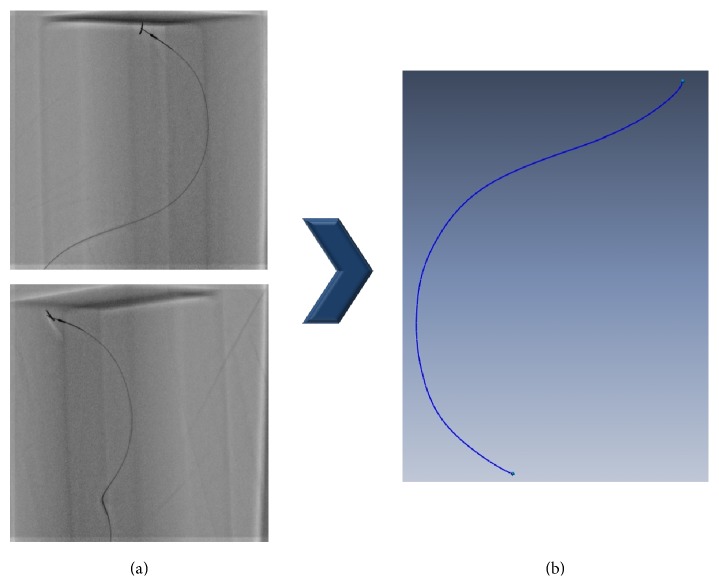
(a) Biplane X-ray angiogram images of IVUS catheter. (b) Restored IVUS catheter path in 3D space.

When taking IVUS images, the path along which the IVUS images are to be taken is secured by inserting a catheter in advance to place an IVUS ultrasonic device at the place where the imaging is to be started. When the IVUS ultrasonic device arrives at the desired position, it follows the catheter and acquires images of the blood vessel cross sections, with the path of the IVUS images matching the path of the catheter. To obtain the catheter path, X-ray angiograms were taken from different directions immediately before the IVUS ultrasonic device was pulled back to take images. The 3D catheter path was generated as shown in Figure 7 using the two 2D catheter paths extracted from the biplane X-ray angiogram images.

### 4.3. Calculation of IVUS Image Position and Orientation

When IVUS images are acquired, the IVUS ultrasonic device moves out of the catheter at a constant speed using the IVUS pullback device. Accordingly, if the 3D catheter path restored using the biplane X-ray angiogram images is divided into as many parts as the number of the IVUS images using the same interval, the positions where the IVUS images have been acquired can be easily calculated. However, as the IVUS ultrasonic device rotates around the catheter when it travels around a bent blood vessel, the IVUS image acquired at this time is in a rotated state.

**Figure 8:**
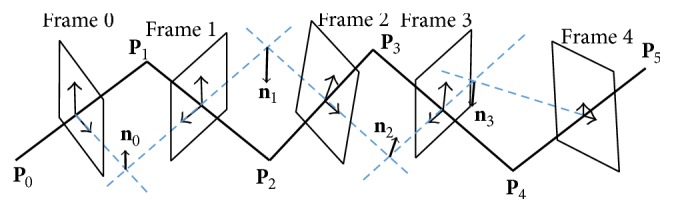
Sequential triangulation method [15].

Whale has proposed the sequential triangulation method that can determine the twist angles of IVUS images using the characteristics of such IVUS images. With this method, the orientations of IVUS images were calculated using only the geometric shape of the catheter restored in 3D. A 3D catheter path was divided into small pieces assuming that it is comprised of innumerable joints and links. The positions and orientations of IVUS images were determined as shown in Figure 8 using the 3 consecutive points on the 3D path divided into smaller pieces. The orientation of each IVUS image is determined by the plane made of the 3 consecutive points existing on the catheter. **P** is the position of each point, and **S**, which is the position of an IVUS image, is the center of the two points as shown in the following [12–17]:

(1) Si=Pi+Pi+12,Si+1=Pi+1+Pi+22.

Also, the tangent vector $\vec{t}$ at **P** is calculated as follows:

(2) t→i=Pi+1−Pi,t→i+1=Pi+2−Pi+1.

The normal vector $\vec{n}$, which is each of the *y*-axis directions of the 2D IVUS images, was calculated by calculating the outer products of the two neighboring tangent vectors $\vec{t}$:

(3) $$\begin{array}{c} \vec{n}={\vec{t}}_{i}\times {\vec{t}}_{i+1}. \end{array}$$

**Figure 9:**
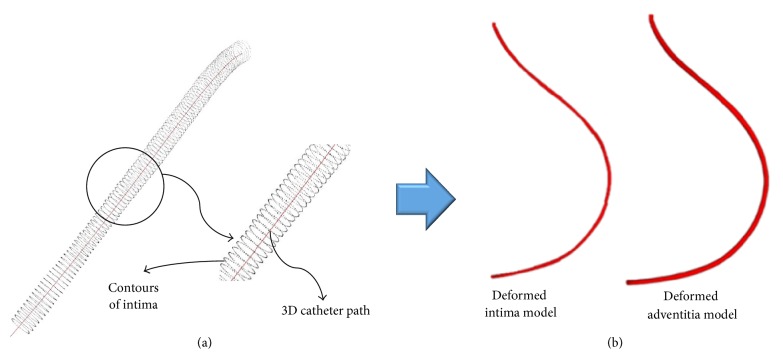
(a) A series of deformed intima cross sections. (b) A polygon model of deformed intima and adventitia model.

Through such a method, the position and orientation where an IVUS image was taken were determined from the 3D path of the catheter.  Figure 9(a) shows the result of applying the position and orientation calculated using the sequential triangulation method to the cross sections of the blood vessel intima and adventitia extracted from a 2D IVUS image, and Figure 9(b) shows the polygon model generated using the points in 3D space. As these models were generated by combining the IVUS and biplane X-ray angiogram images taken in a state deformed by a catheter, they are the blood vessel intima and adventitia models deformed by insertion of a catheter.

## 5. Computation of Undeformed Intima and Adventitia Model by Registration

In this chapter, we intend to compute a 3D intima and adventitia model without the catheter-induced deformation. To achieve this, the 3D model of deformed intima and adventitia generated by combining the IVUS and biplane X-ray angiogram images was registered with the 3D model of the undeformed intima generated using a CT image. As these two 3D models do not only exist on different coordinate systems but also have different scales, there are difficulties in directly registering these 3D models. Accordingly, in this study, we propose a method of determining the corresponding relation between the two 3D blood vessel models to extract the cross sections at the corresponding positions and matching them.

### 5.1. Calculation of Centerline and Extraction of Cross Section

To define a plane required for extraction of 2D cross sections from the 3D blood vessel intima model in a tube form, one 3D point and normal vector are required. For this, the centerline that could well express the shape of the blood vessel should be calculated.

In the study carried out by Luca, the centerline existing between two points within a model in a tube form was defined to be the line farthermost from the boundary. Accordingly, the centerline of an object **Ω** existing in a 3D space can be expressed as the path **C** = **C**(**s**) between two points **P**_1_ and **P**_2_ which minimizes

(4) $$\begin{array}{c} E_{\text{centerline}}\left(\;C\;\right)=\overset{L=C^{-1}\left(P_{1}\right)}{\underset{0=C^{-1}\left(P_{0}\right)}{\int }}F\left(\;C\left(\;s\;\right)\;\right)ds. \end{array}$$

For this, the Delaunay triangulation of the object **Ω** was calculated, through which the maximum spheres inscribed in the blood vessel model were calculated. The centerline of the 3D blood vessel model was extracted using the center points of these spheres.

### 5.2. Correspondence Definition between 3D Blood Vessel Models and Extraction of Cross Sections

To register two blood vessel models in different states, correspondence between the two models should be defined first. For this, the centerlines of the two intima models calculated earlier were used. Because the CT, IVUS, and biplane X-ray angiogram images were all obtained by imaging the same section of the blood vessel replica, the 3D blood vessel models generated earlier model the same section of the blood vessel though they are in different states. Accordingly, the corresponding relation between these two intima models was defined by dividing the center curves of these two intima models into the same number of lines using the same interval, and the cross sections of the 3D models were extracted at the defined positions.

### 5.3. Registering between Cross Sections in Different States

In this study, we intend to generate a 3D intima and adventitia model from which the catheter-induced deformation is removed through registration. Accordingly, we attempted to convert the cross sections of the deformed intima and adventitia extracted earlier into the cross sections of the undeformed intima and adventitia. For this, the cross sections of the deformed intima and adventitia were registered with the cross sections of the undeformed intima.

Registration is the calculation of the coordinate transformation that can minimize the distance between two point sets. Accordingly, registration in this study is to calculate the translation (*x*, *y*), rotation (*θ*), and scale (*s*) that minimizes the distance between the two point sets (**X**: target point cloud, **Y**: source point cloud), which compose the 2D blood vessel cross sections. In this study, the coordinate transformation matrix **T**_0_ that minimizes the distance between the two point sets **X** and **Y** was calculated using the optimization method after setting these 4 elements as the variables. In addition, to make a result linear to the rotation value of the previous frame when registering cross sections, the value closest to the rotation value *θ* of the previous frame was calculated.

(5) $$\begin{array}{c} T_{0}=\min\left(\;\sum \mathrm{dist}\left(\;X,Y^{\prime }\;\right)\;\right), \end{array}$$

where

(6) $$\begin{array}{c} Y^{\prime }=T\left(\;x,y,\theta ,s\;\right)Y. \end{array}$$

**Figure 10:**
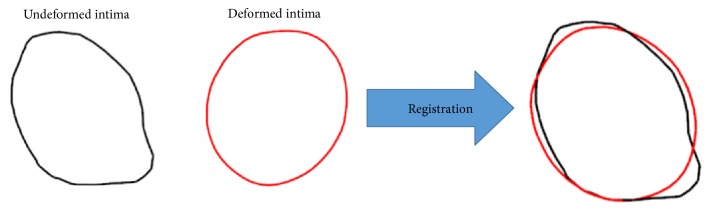
Registration between undeformed and deformed intima contours using the proposed method.

To achieve this, the multiminimizer function of the GNU Scientific Library was used [18].  Figure 10 shows the registration result of the two intima cross sections.

**Figure 11:**
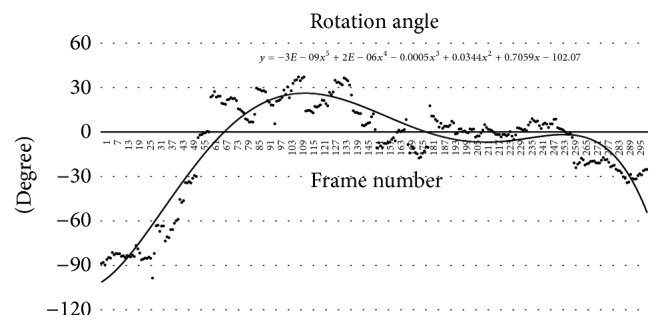
Trend line of rotation angle result.

The *x*, *y*, *θ*, and *s* calculated through the registration between intima cross sections are the values at which the deformed intima cross section changes to the undeformed intima cross section. Accordingly, the calculated *x*, *y*, *θ*, and *s* were equally applied to change the deformed adventitia cross section to the undeformed adventitia cross section.  Figure 11 shows the rotation values of all the cross sections registered using the optimization method. To more linearly transform such rotation values, the trend line was calculated using all the rotation values, and the rotation value of each cross section was corrected to the trend line value.

### 5.4. Generation of an Undeformed Intima and Adventitia Model

**Figure 12:**
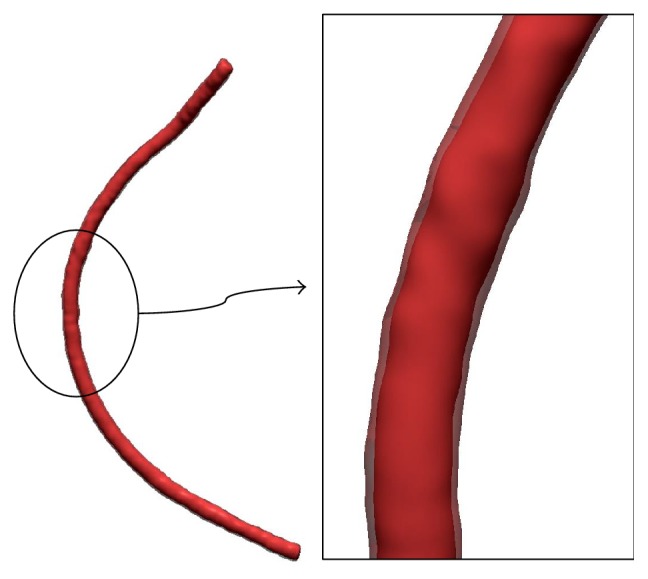
Generated 3D blood vessel model including intima and adventitia.

The cross sections of the undeformed intima and adventitia were calculated through a process similar to that above. To finally generate a model in an undeformed state using such cross sections, the cross sections should be located at the proper positions and in proper orientation. For this, the centerline extracted from the 3D model of the intima not deformed by a catheter, which was generated from a CT image, was used. A 3D blood vessel polygon model, which included the intima and adventitia as shown in Figure 12, was generated by placing the calculated cross sections of the undeformed intima and adventitia on the undeformed centerline.

## 6. Bifurcated Blood Vessel Model

**Figure 13:**
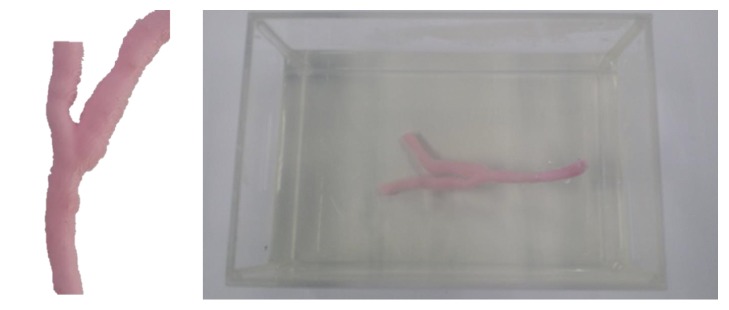
Replica of blood vessel.

In fact, human blood vessels are not comprised of single blood vessels but a combination of blood vessels with many branches. Accordingly, to actually model the blood vessel of a patient, not a single blood vessel model but a 3D blood vessel model that includes branches should be generated. Accordingly, a 3D blood vessel model including branches not deformed by a catheter was generated using the proposed blood vessel modeling method in this chapter. For this, a blood vessel replica including branches was produced as shown in Figure 13. Different from the case of a single blood vessel, this replica was produced by creating a 3D model using the CT images of an actual patient and producing a mold using a 3D printer. A blood vessel replica of the desired form was produced by injecting silicon into this mold. For this replica, gelatin was again used to fix the blood vessel tube.

**Figure 14:**
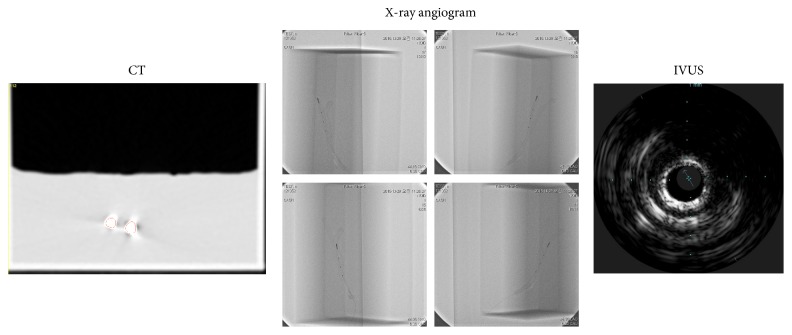
CT, biplane X-ray angiogram, and IVUS images of replica.

The CT, IVUS, and biplane X-ray angiogram images were taken using the produced blood vessel replica as per the case of a single blood vessel. As the replica includes branches, the IVUS images and the X-ray angiogram images of each blood vessel branch were taken.  Figure 14 shows the medical images taken using the blood vessel replica.

### 6.1. Generation of a 3D Intima Model Including Branches Not Deformed by a Catheter and Extraction of Cross Sections

**Figure 15:**
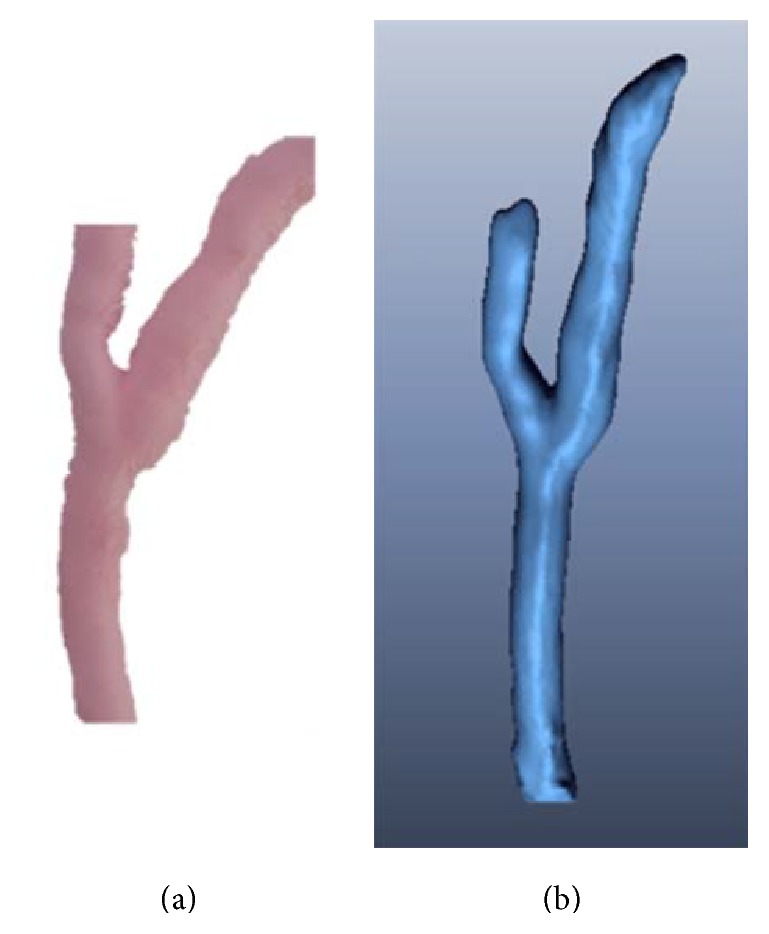
(a) Bifurcated artificial blood vessel model. (b) Generated undeformed 3D intima model.

In the case of the blood vessel that includes branches, a 3D model of the intima not deformed by a catheter was also generated using the CT image as per the single blood vessel.  Figure 15 shows the 3D model of the intima not deformed by a catheter, which was generated using a CT image.

**Figure 16:**
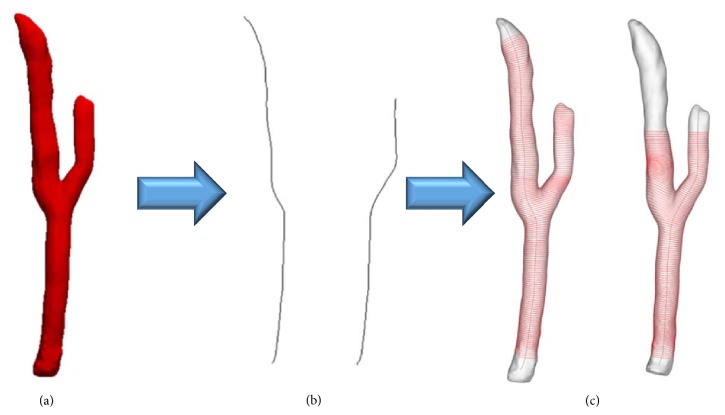
(a) Undeformed intima model. (b) Centerlines of each branch. (c) Extracted cross sections using each centerline.

To extract the cross sections of the 3D intima models, the centerline of each branch was calculated using a 3D Voronoi diagram.  Figure 16 shows the centerline of each branch and the cross sections extracted using them.

### 6.2. Generation of a 3D Model of the Intima and Adventitia Not Deformed by a Catheter That Includes Branches and Extraction of Cross Sections

**Figure 17:**
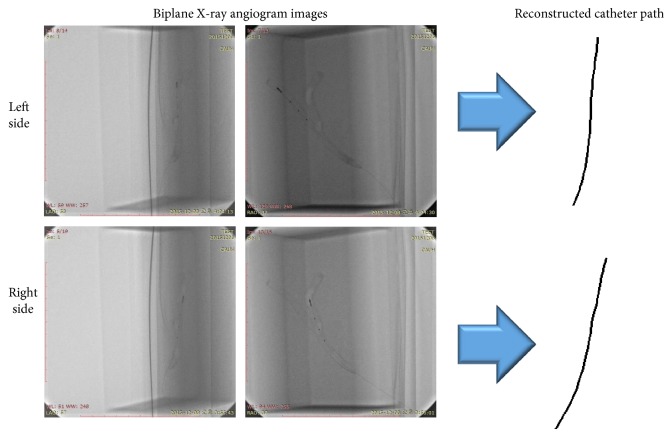
Reconstructed 3D catheter path of each branch.

To generate a blood vessel model that includes branches, the IVUS images of all the blood vessel branches should be taken to obtain data about the intima and adventitia of each blood vessel branch. In addition, to calculate the position and orientation of the IVUS image of each branch, when the IVUS image of each blood vessel branch is taken, the inserted catheter should be photographed from different directions. Accordingly, as the blood vessel replica used in this study had two blood vessel branches, 2 sets of biplane X-ray angiogram images were acquired by photographing the catheter inserted into each blood vessel branch twice from different directions, which were used to generate two 3D paths of the catheter as shown in Figure 17.

**Figure 18:**
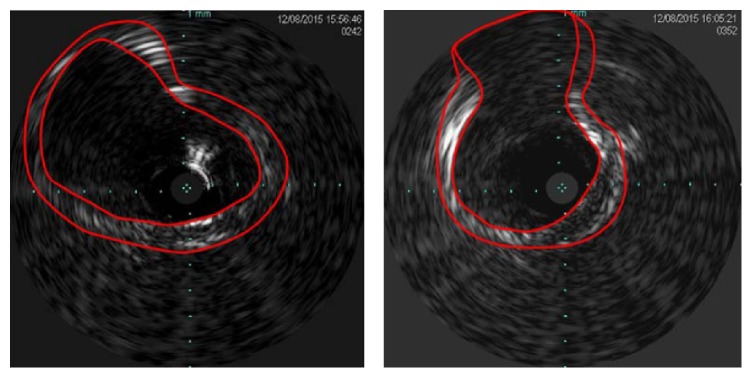
Segmented intima and adventitia contours from IVUS image at bifurcation.

In addition, to acquire the detailed shape of the blood vessel, the two sets of IVUS images obtained by imaging each blood vessel branch were used. In the case that branches are included, as in the case of the CT images, the IVUS images also show the sections where the blood vessel is bifurcated as shown in Figure 18. When the cross sections in the IVUS images were registered with the cross sections extracted from the CT images, the cross sections of the relevant intima and adventitia were all extracted from the IVUS images so that the branched sections can be accurately matched. Furthermore, even when the shapes of the blood vessel intima and adventitia on the other side are not perfectly obtained, the shapes of the intima and adventitia were extracted by overlapping them as shown in Figure 18.

**Figure 19:**
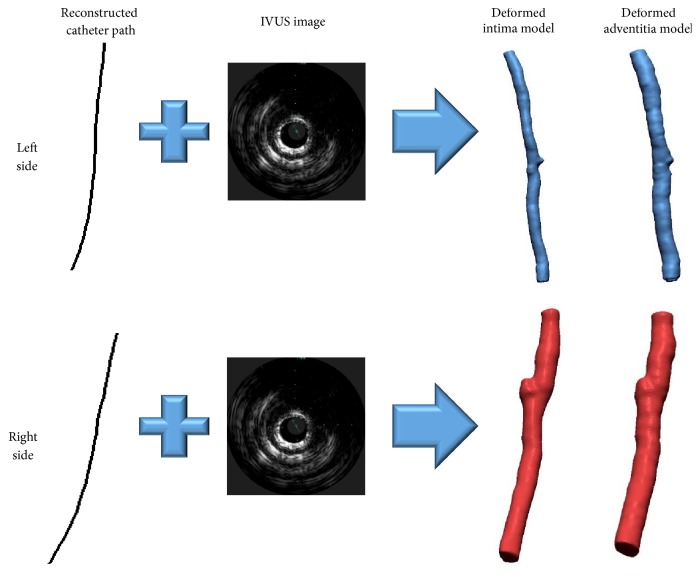
Reconstructed deformed 3D intima and adventitia models of each branch.

A 3D model of the intima and adventitia not deformed by the catheter inserted was generated as shown in Figure 19 by applying the result of the sequential triangulation method using each 3D catheter path to each cross section of the intima and adventitia extracted from the IVUS images.

### 6.3. Computation of a 3D Model of the Intima and Adventitia Including Branches with the Deformation Caused by the Catheter Eliminated through Registration

**Figure 20:**
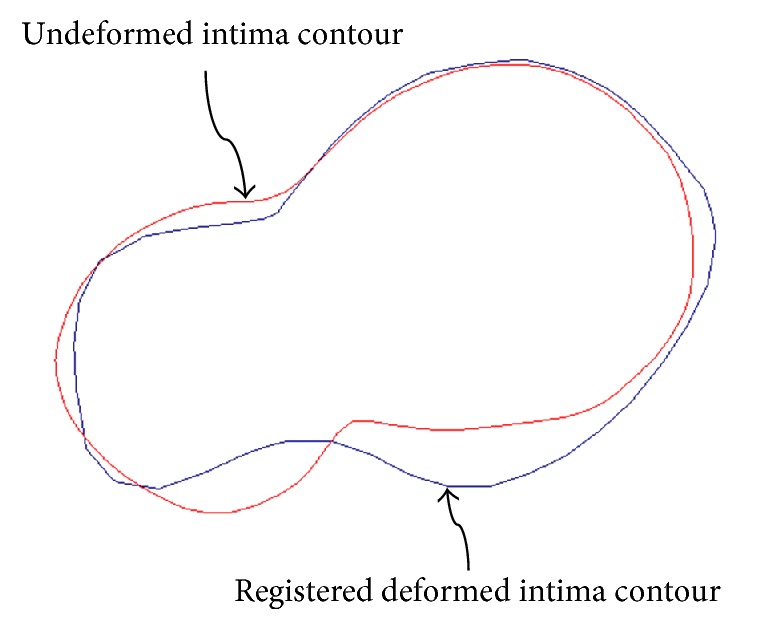
Comparison undeformed intima contour with registered deformed intima contour.

In the case of a blood vessel that includes bifurcation, a 3D model of intima and adventitia deformed by a catheter is generated in the form of a single blood vessel for each branch, and the cross sections are also found to be similar to the case of a single blood vessel. Accordingly, to carry out registration using these cross sections, the intima cross sections extracted from each branch in the undeformed intima model, which had been generated through the CT images earlier, were used directly. The cross sections of the undeformed intima and adventitia were calculated by registering the cross sections of the deformed intima and adventitia with the cross sections of the undeformed intima that included these branch points.  Figure 20 shows the result of registration between the cross sections of the undeformed and deformed intima at a branch point. It can be seen that even when branch points are included, cross sections can be properly matched using the proposed registration method in this study.

**Figure 21:**
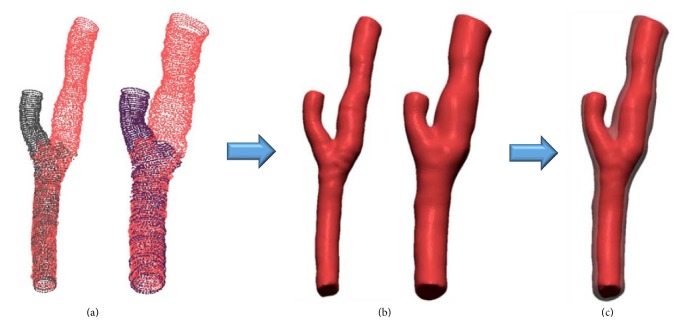
(a) Intima and adventitia point sets placed on undeformed centerline. (b) Computed undeformed intima and adventitia model. (c) Three-dimensional blood vessel model including intima and adventitia.

The rotation values of the registered cross sections were corrected to enable the rotation variations of the cross sections to be linear using the trend line equations of the rotation values of the cross sections when all the cross sections of the right and left blood vessel branches are registered. After transforming the cross sections of the intima and adventitia in a deformed state into the cross sections of the intima and adventitia in an undeformed state through such a registration process, all the cross sections were placed on the centerline extracted from the 3D model in an undeformed state as shown in Figure 21(a). A model of the intima and the adventitia that included a branch point was generated as shown in Figure 21(b), using all the points corresponding to the left and right blood vessel branches, which were used to generate a 3D blood vessel model that included intima and adventitia.

## 7. Conclusion and Discussion

In this paper, we have proposed a method for generating a 3D model of intima and adventitia for accurate FSI analysis that eliminates the deformation caused by insertion of a catheter. The method of combining IVUS images and biplane X-ray angiogram images is widely used for generation of 3D blood vessel models and generates a 3D model of the intima and adventitia that is deformed by the inserted catheter. To eliminate such deformation, a 3D model of the intima without catheter-induced deformation was additionally generated from CT images, and these two models were registered to eliminate the catheter-induced deformation.

In the registration, the 3D models were not directly registered but the cross sections of each model were registered. The cross sections of the deformed intima were registered with the cross sections of the undeformed intima, and the cross sections of the undeformed adventitia were converted by applying the registration result to the cross sections of the deformed adventitia. A 3D blood vessel model that included the undeformed intima and adventitia was finally generated by placing the cross sections of the undeformed intima and adventitia calculated through such a process on the centerline extracted from the undeformed intima model.

The method of modeling a 3D blood vessel proposed in this study has various limitations. To determine the position and direction of the intima and adventitia cross sections extracted from IVUS images, these cross sections were registered with the cross sections of the intima extracted from CT images. The values of movement (*x*, *y*), rotation (*θ*), and scale (*s*) calculated through the registration between the two intima cross sections were equally applied to the cross sections of the adventitia extracted from IVUS images. However, such a method calculates an ideal result without considering the material properties of the blood vessel. In the case of an actual blood vessel, the intima and the adventitia will not equally deform because of the material properties of the blood vessel wall. In addition, for a patient with atherosclerosis, the blood vessel wall will not be isotropic owing to the plague existing on the blood vessel wall. Accordingly, the intima and adventitia model calculated using the method proposed in this study contains such errors.

Another limitation is that it is difficult to accurately evaluate the accuracy of the blood vessel model generated through the proposed method. This is because the only medical image through which the information about blood vessel adventitia can be obtained is IVUS image.

If OCT (Optical Coherence Tomography) that can photograph lumen more clearly than IVUS is used to further this study, more accurate information about blood vessel intima can be obtained. However, as OCT uses light, there are difficulties in obtaining accurate information about adventitia unlike IVUS that uses ultrasound. Accordingly, more precise 3D blood vessel models are expected to be generated by using OCT to obtain intima data and IVUS to obtain adventitia data.

## References

[1] Dumont K., Vierendeels J., Kaminsky R., Van Nooten G., Verdonck P., Bluestein D. (2007). Comparison of the hemodynamic and thrombogenic performance of two bileaflet mechanical heart valves using a CFD/FSI model. *Journal of Biomechanical Engineering*.

[2] Reymond P., Crosetto P., Deparis S., Quarteroni A., Stergiopulos N. (2013). Physiological simulation of blood flow in the aorta: comparison of hemodynamic indices as predicted by 3-D FSI, 3-D rigid wall and 1-D models. *Medical Engineering and Physics*.

[3] Huang X., Yang C., Zheng J. (2014). Higher critical plaque wall stress in patients who died of coronary artery disease compared with those who died of other causes: a 3D FSI study based on ex vivo MRI of coronary plaques. *Journal of Biomechanics*.

[4] Pakravan H. A., Saidi M. S., Firoozabadi B. (2015). FSI simulation of a healthy coronary bifurcation for studying the mechanical stimuli of endothelial cells under different physiological conditions. *Journal of Mechanics in Medicine and Biology*.

[5] Valenciaa A., Muñoza F., Arayaa S., Riverab R., Bravob E. (2009). Comparison between computational fluid dynamics, fluid–structure interaction and computational structural dynamics predictions of flow-induced wall mechanics in an anatomically realistic cerebral aneurysm model. *International Journal of Computational Fluid Dynamics*.

[6] Knight J., Baumüller S., Kurtcuoglu V. (2010). Long-term follow-up, computed tomography, and computational fluid dynamics of the Cabrol procedure. *Journal of Thoracic and Cardiovascular Surgery*.

[7] Qian Y., Liu J. L., Itatani K., Miyaji K., Umezu M. (2010). Computational hemodynamic analysis in congenital heart disease: simulation of the Norwood procedure. *Annals of Biomedical Engineering*.

[8] Tse K. M., Chiu P., Lee H. P., Ho P. (2011). Investigation of hemodynamics in the development of dissecting aneurysm within patient-specific dissecting aneurismal aortas using computational fluid dynamics (CFD) simulations. *Journal of Biomechanics*.

[9] Lee W., Ryou H. S., Kim S., Nam J. W., Lee W. S., Cho S. W. (2015). Study of hemodynamic parameters to predict coronary artery disease using assumed healthy arterial models. *Journal of Mechanical Science and Technology*.

[10] Antiga L. (2002). *Patient-Specific Modeling of Geometry and Blood Flow in Large Arteries*.

[11] Antiga L., Ene-Iordache B., Remuzzi A. (2003). Computational geometry for patient-specific reconstruction and meshing of blood vessels from MR and CT angiography. *IEEE Transactions on Medical Imaging*.

[12] Wahle A., Oswald H., Fleck E. (1993). New 3-D attributed data model for archiving and interchanging of coronary vessel systems. *Computers in Cardiology*.

[13] Wahle A., Wellnhofer E., Mugaragu I., Sauer H. U., Oswald H., Fleck E. Quantitative volume analysis of coronary vessel systems by 3-D reconstruction from biplane angiograms.

[14] Wahle A., Wellnhofer E., Mugaragu I., Sauer H. U., Oswald H., Fleck E. (1995). Assessment of diffuse coronary artery disease by quantitative analysis of coronary morphology based upon 3-d reconstruction from biplane angiograms. *IEEE Transactions on Medical Imaging*.

[15] Wahle A., Prause G. P. M., DeJong S. C., Sonka M. (1998). 3-D fusion of biplane angiography and intravascular ultrasound for accurate visualization and volumetry. *Medical Image Computing and Computer-Assisted Intervention—MICCAI '98: First International Conference Cambridge, MA, USA, October 11–13, 1998 Proceedings*.

[16] Wähle A. (1999). Geometrically correct 3-D reconstruction of intravascular ultrasound images by fusion with biplane angiography-methods and validation. *IEEE Transactions on Medical Imaging*.

[17] Wahle A., Mitchell S. C., Olszewski M. E., Long R. M., Sonka M. Accurate visualization and quantification of coronary vasculature by 3-D/4-D fusion from biplane angiography and intravascular ultrasound.

[18] Gough B. (2009). *GNU Scientific Library Reference Manual*.

[19] Schoenhagen P., Nissen S. E., Murat E. (2005). *IVUS Made Easy*.

